# Aryl hydrocarbon receptor and Krüppel like factor 10 mediate a transcriptional axis modulating immune homeostasis in mosquitoes

**DOI:** 10.1038/s41598-022-09817-2

**Published:** 2022-04-09

**Authors:** Aditi Kulkarni, Ashmita Pandey, Patrick Trainor, Samantha Carlisle, Wanqin Yu, Phanidhar Kukutla, Jiannong Xu

**Affiliations:** 1 Biology Department, New Mexico State University, Las Cruces, NM 88003 USA; 2 Department of Chemistry and Biochemistry, New Mexico State University, Las Cruces, NM 88003 USA

**Keywords:** Immunology, Microbiology, Molecular biology

## Abstract

Immune responses require delicate controls to maintain homeostasis while executing effective defense. Aryl hydrocarbon receptor (AhR) is a ligand-activated transcription factor. The Krüppel-like factor 10 (KLF10) is a C2H2 zinc-finger containing transcription factor. The functions of mosquito AhR and KLF10 have not been characterized. Here we show that AhR and KLF10 constitute a transcriptional axis to modulate immune responses in mosquito *Anopheles gambiae*. The manipulation of AhR activities via agonists or antagonists repressed or enhanced the mosquito antibacterial immunity, respectively. KLF10 was recognized as one of the AhR target genes in the context. Phenotypically, silencing *KLF10* reversed the immune suppression caused by the AhR agonist. The transcriptome comparison revealed that silencing *AhR* and *KLF10* plus challenge altered the expression of 2245 genes in the same way. The results suggest that KLF10 is downstream of AhR in a transcriptional network responsible for immunomodulation. This AhR–KLF10 axis regulates a set of genes involved in metabolism and circadian rhythms in the context. The axis was required to suppress the adverse effect caused by the overactivation of the immune pathway IMD via the inhibitor gene *Caspar* silencing without a bacterial challenge*.* These results demonstrate that the AhR–KLF10 axis mediates an immunoregulatory transcriptional network as a negative loop to maintain immune homeostasis.

## Introduction

Mosquitoes, like many insects, have evolved an efficient innate immune system consisting of Toll, IMD, JAK-STAT, and RNAi pathways to defend against infection with bacteria, fungi, viruses, and parasites^1–4^. During evolution, various regulatory circuits have evolved in insects to execute an effective defense and avoid the deleterious effects of overaggressive immune responses and immunopathogenesis^5^. It has been well documented that intrinsic negative regulators Cactus, Caspar, and SOCS for the Toll, IMD, and Jak-STAT pathways, respectively, delicately tether the immune pathways. Besides, chemical defense mechanisms play important roles in homeostasis. Xenobiotic sensors are located at the interface to detect the chemicals that originated from the associated microbiota or environment. These sensors recognize ligands and initiate responses to coordinate context-dependent xenobiotic metabolism and immune defenses^6^. The aryl hydrocarbon receptor (AhR) is a ligand-activated transcription factor, which was identified in the 1970s as a xenobiotic sensor recognizing the compound 2,3,7,8-tetrachlorodibenzo-p-dioxin (TCDD) in mice. Based on the studies over the past five decades, it is well known that AhR can recognize various endogenous and exogenous ligands^7^ and mediate transcription of target genes participating in multiple physiological processes, including immune regulation and xenobiotic metabolism^8,9^. AhR is an ancient protein with an ortholog identified in the placozoan *Trichoplax* and conserved up to humans^9,10^. According to the studies in the fruit fly *Drosophila melanogaster*, the nematode *Caenorhabditis elegans,* and the cnidarian *Nematostella vectensis*, the ancestral functions of AhR are related to the development of sensory structures as well as neural systems^10^. These ancestral functions are likely mediated by endogenous ligands. During evolution, as an add-on function, AhR has further acquired the capability to recognize ligands derived from exogenous chemicals^9,10^. Vertebrate AhR is a critical player in coordinating transcriptional circuits for immune regulation upon recognition of certain xenobiotics^11^. The connections between a xenobiotic sensor and immune regulation unveil the role of chemical sensing systems in transducing non-self signals into proper responses to execute effective immune defense while maintaining homeostasis. In contrast to the rich studies about the diverse functions of AhR in the vertebrates, very little is known about the AhR in insects except for its role in development. Recently, Sonowal et al. showed that AhR in fruit fly and nematodes can recognize indoles derived from symbiotic bacteria and initiate transcription of a gene set contributing to healthspan in the flies and nematodes^12^. In this study, we demonstrate that AhR and Krüppel-like factor 10 are connected to mediate a transcriptional axis for immune modulation, which suggests that at insect level AhR has acquired functions to modulate innate immune responses.

## Results

### Manipulation of AhR activity affects immunity against bacterial infection in mosquitoes

**Figure 1:**
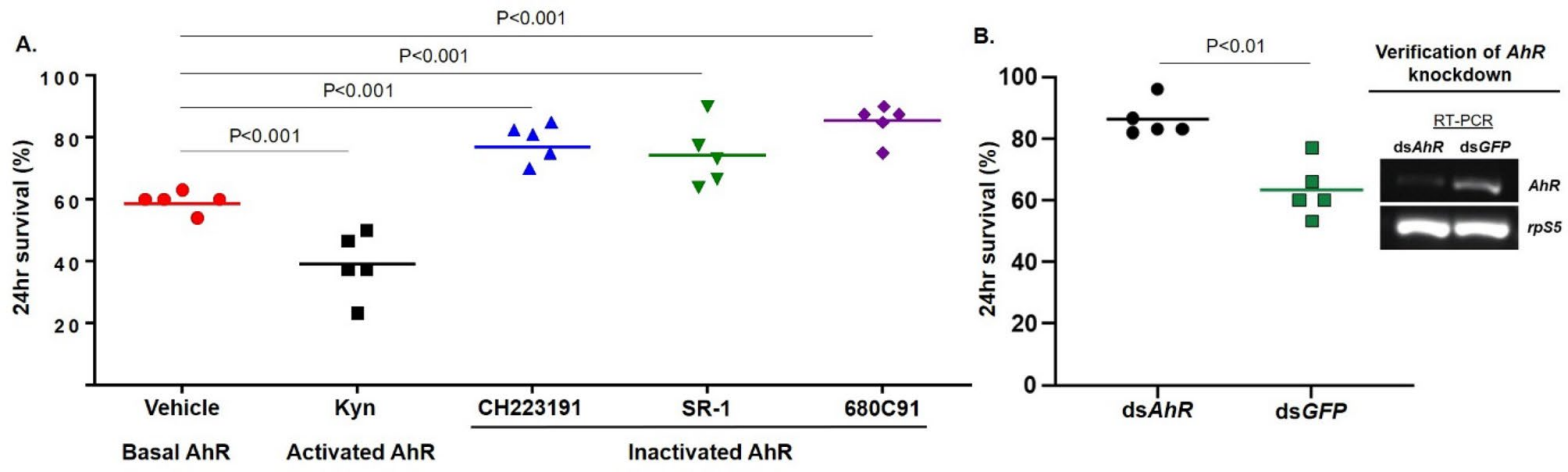
Mosquito survival upon bacterial challenge when AhR activity is manipulated pharmacologically or genetically. (**A**) The effect of pharmacological manipulation of AhR activity on the survival post *Serratia* challenge. Average 24 h-survival (%) was denoted by the line. (**B**) The effect of *AhR* silencing on the survival post-challenge. The *AhR* knockdown was verified by RT-PCR. The 24 h-survival data were generated from 5 replicates and tested by Chi-square.

AhR is conserved from invertebrates to mammals. AhRs from mosquitoes *An. gambiae* and *Aedes aegypti* were clustered with the AhRs from the fruit fly *D. melanogaster* and nematode *C. elegans* in the same clade, while AhRs from zebrafish *Danio rerio,* mouse *Mus musculus,* and human *Homo sapiens* were grouped together (Fig. S1A). The bHLH and PAS A domains are involved in DNA binding and dimerization with cofactors, which are more conserved compared to PAS B domain that is involved in ligand binding (Fig. S1B). The higher level of divergence in PAS B suggests diversity in ligand recognition. *AhR* transcripts were detected in the whole body of larvae, pupae, and adults (Fig. S1C), indicating that *AhR* is constitutively expressed in all life forms of the mosquito. To investigate the immune regulatory role of AhR, we pharmacologically manipulated AhR activity and then examined the infection outcomes in mosquitoes upon a bacterial infection. The mosquito innate immune system is responsive to bacterial challenges. Antibacterial immunity has been used as a model to study mosquito innate immunity^4^. Recently we have shown that the bacterium *Serratia fonticola* S1 strain, once injected into the hemocoel intrathoracically, can cause acute hemocoel infection and trigger an immune response. Survival at 24 h post-injection is an informative measure of infection and immune outcomes^13^. Kynurenine (Kyn) is an endogenous AhR ligand^14^, which is an intermediate metabolite of tryptophan oxidation catalyzed by tryptophan-2,3-dioxygenase (TDO) in mosquitoes^15^. TDO can be inhibited by chemical compound 680C91^16^, which leads to the reduction of endogenous Kyn production. CH223191^17^ and SR1^16^ are known AhR antagonists. After pharmacological AhR manipulation through feeding respective chemicals in sugar diet, the mosquitoes were challenged by injecting *Serratia* into the hemocoel, and the 24 h-survival rate was recorded. As shown in Fig. 1A, the vehicle control with a basal AhR activity showed a 58.6% survival. The AhR agonist Kyn reduced the survival to 38.9%. In contrast, the AhR antagonists CH233191, SR1, or 680C91 increased the survival to 75.7–85.4%. This phenotypic pattern was corroborated by the genetic manipulation of AhR. The RNAi-mediated *AhR* knockdown increased survival to 86.3% from the 63.3% in the dsGFP control (Fig. 1B).

### Transcriptomic alterations upon AhR manipulation

To identify genes that are regulated by AhR upon the bacterial infection, we conducted RNA sequencing (RNA-seq) to profile the transcriptomes upon treatment. Mosquitoes were fed with AhR antagonist SR1 or vehicle control and then challenged with *Serratia*. Sterile water injection was used as injury control. Surviving mosquitoes at 24 h post-challenge were processed for RNA-seq. The transcriptional abundance of genes was measured using normalized read counts, and differentially expressed genes were identified by DESeq2. Between the *Serratia* infection and injury control, 2102 genes were differentially expressed (using a cutoff of q-values < 0.05, Table S3), which we defined as infection responsive genes. Among these genes, 265 were upregulated and 145 were downregulated at least twofold. The infection inducible gene set included typical immune genes, such as immune pathway components (e.g., spaetzle1, spaetzle3, PGRPLB, Rel1, and Rel2), immune effectors (e.g., DEFs, CECs, TEPs and LRIMs, CLIP serine proteases, fibrinogens), and immune regulators, such as serpins and inhibitors of apoptosis (IAPs). The AhR antagonist affected the expression of 696 infection-responsive genes, among them 66 upregulated and 36 downregulated genes had at least twofold differences. The AhR antagonist-affected genes were largely unannotated, and the major antimicrobial genes, such as *Def1* and *Cec1*, were not affected, but the genes with potential immune-suppressive functions, such as *IAP4, SRPN6, SRPN10* and *SOCS* require the presence of AhR for the response to infection (Table S3). The expression patterns of *Def1, Tep15, SRPN10*, and *KLF10* were validated by qRT-PCR (Fig. S2).

### AhR and KLF10 mediate a transcriptional axis in the infection context

**Figure 2:**
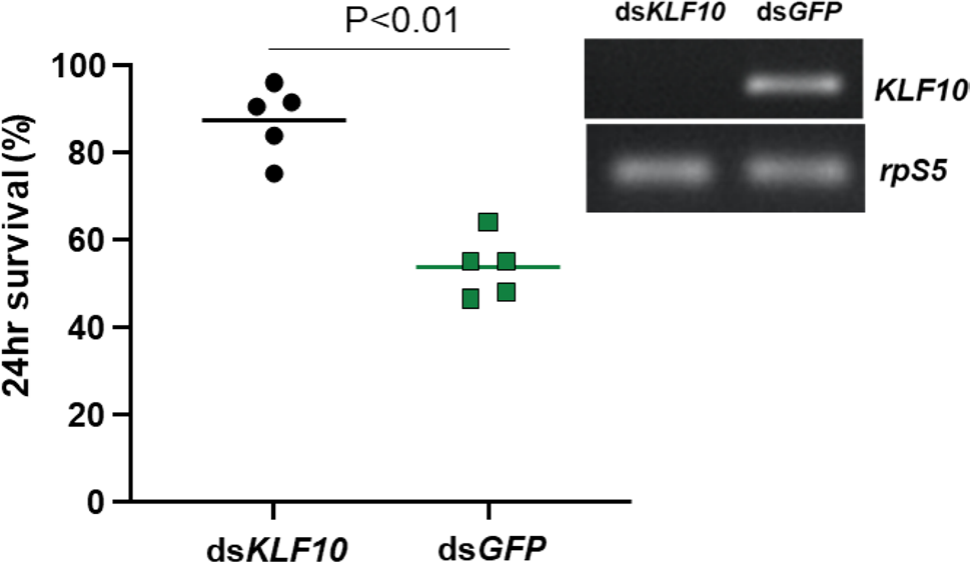
KLF10 affects the survival of bacterial infection. dsKLF10 increased the survival upon *Serratia* challenge. The *KLF10* knockdown was verified by RT-PCR.

**Figure 3:**
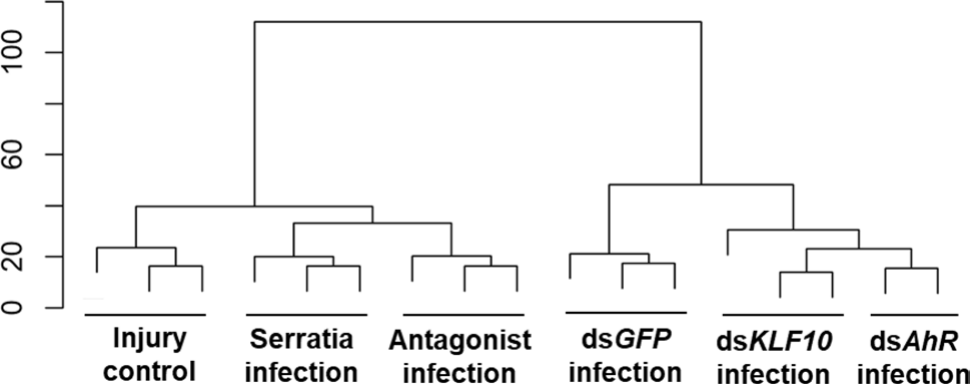
Clustering of transcriptomes in response to *Serratia* challenge. The scale bar represents the distance between the clusters.

**Figure 4:**
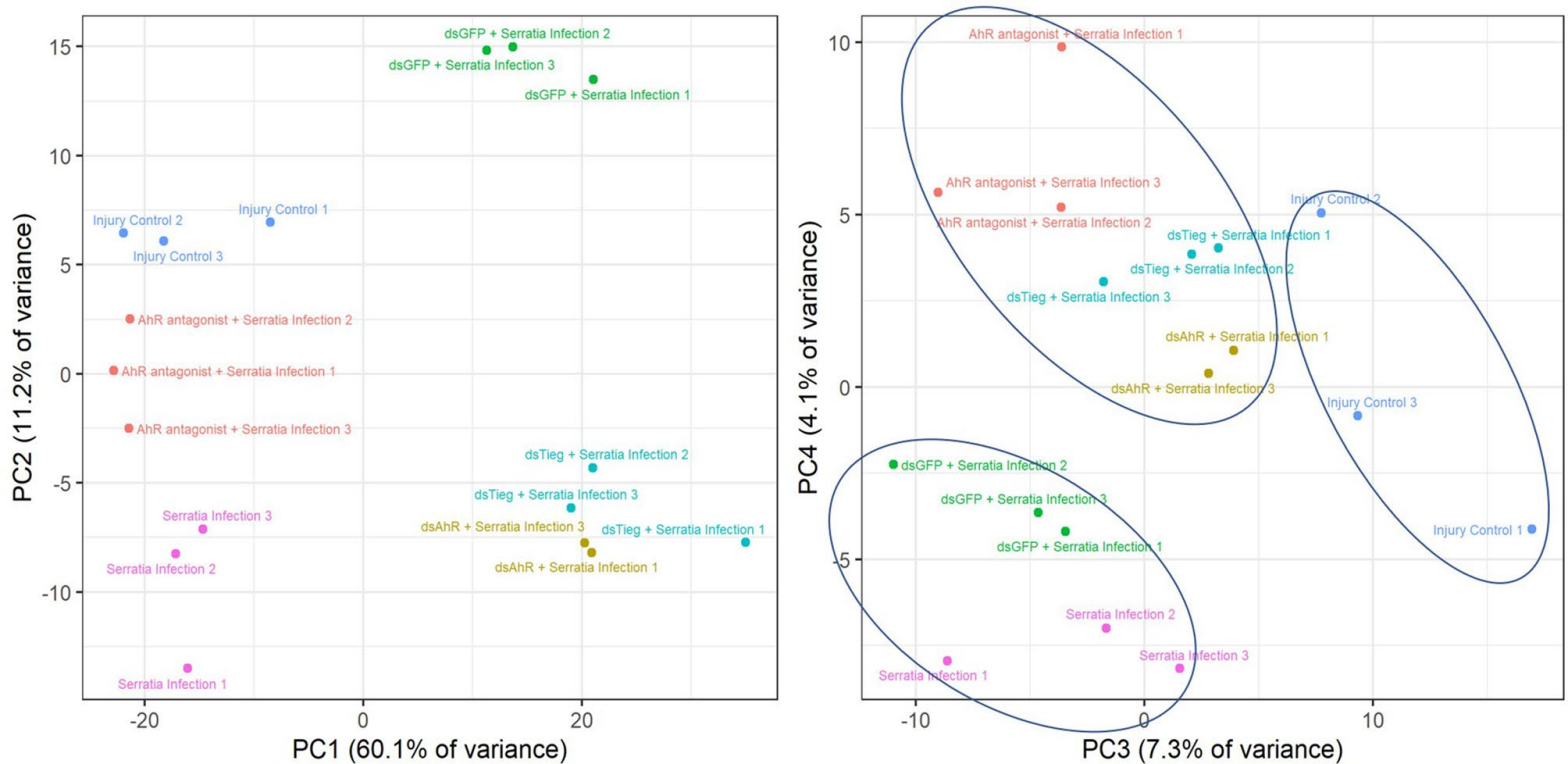
Principal component analysis (PCA) of transcriptomes. (**A**) The PC1 and PC2 explained the major variance of transcriptomes with different treatments. (**B**) The impact of AhR antagonist, dsAhR and dsKLF10 (dsTieg) on the transcriptomic response to the challenge was better represented by PC3 and PC4.

One of the AhR regulated genes, *AGAP009889*, is the ortholog of *cabut* in *Drosophila,* which was named as TGF-β-inducible early-response gene (TIEG)^18^ after the human homolog^19^. *Drosophila* TIEG is involved in the development, metabolism, and growth control^20–23^. TIEG is a C2H2-type zinc finger transcription factor in the Sp1-like/Krüppel-like family, designated as Krüppel-like factor (KLF) 10^24^. TIEG/KLF10 is involved in the TGF-β/Smad signaling^25–27^. AhR and TGF-β/Smad signaling are connected to mediate immune modulation to maintain immune homeostasis in mice^28,29^. Therefore, we tested the hypothesis that KLF10 is downstream of AhR and regulates the transcription of genes responsible for immune modulation. First, the putative AhR binding motif (GCGTG)^30^ was identified in the sequence upstream of the KLF10 coding region, suggesting *KLF10* may be an AhR target gene. Next, we examined the effect of dsKLF10 on the *Serratia* infection outcome. As expected, dsKLF10 resulted in 87.46% of 24 h-survival, higher than 53.72% in the dsGFP control (Chi-square test, P < 0.01, Fig. 2); a pattern similar to the dsAhR effect. Then, we conducted RNA-seq to compare the transcriptomes in response to the dsAhR and dsKLF10 and AhR antagonist. Clustering analysis separated transcriptomes into two clusters (Fig. 3). The injury control was separated from the *Serratia* infection and the antagonist/infection in one cluster. In the other cluster, the dsAhR/infection and the dsKLF10/infection were grouped and distinct from the dsGFP/infection. The PCA analysis revealed that principal component 1 (PC1) and PC2 explained 61% and 11.2% of the variance, respectively, the transcriptome replicates of each treatment were closely clustered, and the transcriptomes of different treatments were separate distinctly (Fig. 4). Replicates of dsAhR and dsKLF10 were located closely, indicating a similarity in their transcriptomic responses. However, the replicates of dsGFP/infection were separate distinctly from the replicates of *Serratia* infection, suggesting that the dsGFP treatment had other effects. In addition, there was an evident separation between the AhR antagonist and the *AhR* silencing, suggesting that each of the two approaches had other effects in addition to affecting AhR activity. The AhR antagonist was administrated via diet, while the dsRNA was administered through injections, an invasive method. In insects, dsRNA triggers defense against viruses^31,32^. The dsGFP injection may have an unknown influence on the response to the following bacterial challenge. Indeed, in response to the *Serratia* challenge, the cohorts with naïve backgrounds and the cohort with dsGFP treatment shared only 450 genes, and the dsGFP cohorts demonstrated 2099 unique responsive genes, and the native cohorts had 481 unique responsive genes (Fig. S3). These confounding effects may contribute to the patterns revealed by PC1 and PC2. The PC3 and PC4 captured 7.3% and 4.1% of the variance, the replicates of AhR-antagonist/infection, dsAhR/infection and dsKLF10/infection were clustered more closely, which separated from the clusters of *Serratia* infection and dsGFP/infection (Fig. 4). Therefore, PC3 and PC4 may represent the effect of AhR manipulation more genuinely and describe more accurately the transcriptomic responses that are driven by the AhR–KLF10 signaling axis. This pattern was further corroborated by co-expression module analysis. The weighted gene correlation network analysis (WGCNA) was implemented to identify phenotype-associated modules of co-expressed genes. WGCNA identified 35 modules from the entire dataset, each containing 23–1382 differentially expressed (DE) genes. The treatment-affected genes were not distributed evenly in these modules. As shown in Fig. S4. Category I includes 9 modules, representing 62.1% (1305/2102) DE genes in the infection cohorts, but only 30.5% (498/1631) in the AhR antagonist cohorts, 30.6% (1007/3285) in the dsAhR cohorts, and 27.2% (888/3260) in the dsKLF10 cohorts. Category II includes 5 modules, representing 8.1% (170/2102) DE genes in the infection cohorts, but 26.3% (429/1631) in the antagonist, 37.2% (1223/3285) in the dsAhR and 36.4% (1185/3260) in the dsKLF10 cohorts. Overall, the modules in Category I and II represent genes targeted by the AhR–KLF10 axis (Fig. S4). Together, the transcriptome comparisons indicate that the two manipulation approaches had resulted in broad impacts on a variety of genes with different functions. The PCA and WGCNA module analysis effectively extracted transcriptomic patterns caused by the AhR manipulations.

**Figure 5:**
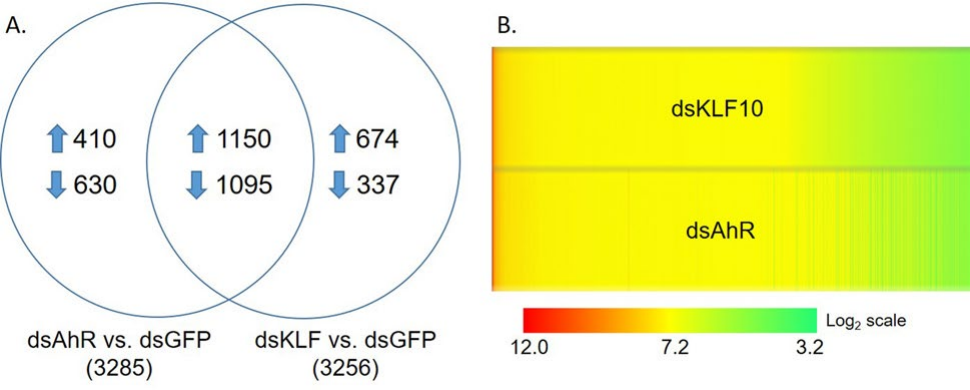
Expression patterns of the genes that were regulated by the AhR–KLF10 axis. (**A**) The distribution of altered genes by respective treatments. (**B**) The heatmap of transcript abundance (TPM) of the genes shared in the dsAhR and dsKLF10 cohorts.

The expression patterns of a majority of genes were consistent between the dsAhR and the dsKLF10 treatments. As shown in Fig. 5A, 3285 genes and 3256 genes in the dsAhR and the dsKLF10 cohorts were altered upon the *Serratia* challenge, respectively. Among them, 2245 genes were shared by both treatments, accounting for 68.3% and 68.9% of the total genes that were affected in the respective cohorts. From the shared genes, 1150 were upregulated and 1095 were downregulated in the context. These genes exhibited the same transcriptional directions in the dsAhR and dsKLF10 cohorts (Fig. 5B). This remarkable pattern indicates that approximately 2/3 of the AhR-regulated genes are the KLF10 target genes as well, supporting the hypothesis that AhR and KLF10 act as a transcriptional axis in response to the infection. Interestingly, in the dsAhR and dsKLF10 altered genes, 720 out of 1150 upregulated genes and 893 out of 1095 downregulated genes were not infection-responsive. In other words, these genes did not transcriptionally respond to the challenge when the axis was present. This suggests that the AhR–KLF10 axis may restrain the transcriptional response of a large number of genes in the infection context. The annotation of these genes suggests that they are involved in various processes (Table S4).

KLF10 can be a transcriptional activator or repressor^33^. This pattern was observed in the mosquitoes as well. In the dsAhR and dsKLF10 cohorts, the expression of 1150 genes was increased and that of 1095 genes was decreased, respectively, indicating that the AhR–KLF10 axis enhances or represses the transcription of these genes. The mammalian KLF10 and Drosophila TIEG/KLF10 (Cabut) are involved in the ChREBP/Mondo-Mlx transcriptional network for sugar sensing and circadian control of metabolic processes^21,34,35^. In this context, KLF10 is a transcriptional repressor of phosphoenolpyruvate carboxykinase (PEPCK). In the mosquitoes, *pepck* was induced by the infection, but this induction was under the repressive control of the AhR–KLF10 axis as the *pepck* expression was increased in both dsAhR and dsKLF10 upon the *Serratia* challenge (Fig. S5). In Drosophila, sugarbabe is a transcription factor in the Mondo-Mlx hierarchical network^36^. *AGAP006736* is the ortholog of *sugarbabe*, its transcript abundance was increased in both dsAhR and dsKLF10 cohorts, suggesting that its expression was repressed by the AhR–KLF10 axis (Fig. S5). The circadian clock orchestrates immunity^37^ as well as metabolism^38^. KLF10 represses the circadian genes in the Mondo-Mlx network^21,35^. In the dsAhR and dsKLF10 cohorts, circadian genes *tim*, *cwo*, and *vri* were upregulated, suggesting they were negatively controlled by the axis (Fig. S5). A set of immune genes such as *Toll5A, Rel2, ClipB19, TEP3* and *TEP15,* were affected by the axis*.* These genes were all induced by the infection, but their transcription level was limited by the axis. When the axis was repressed by knockdown, their expression was higher (Fig. S5). The axis also acts as an activator to promote the transcription of certain genes, such as Mondo-Mlx target genes *arrestin 1* and *2*, *G-protein coupled receptors* (*GPRop1* and *3*), and *vitellogenin receptor,* their expression was downregulated by silencing the axis (Fig. S5).

**Figure 6:**
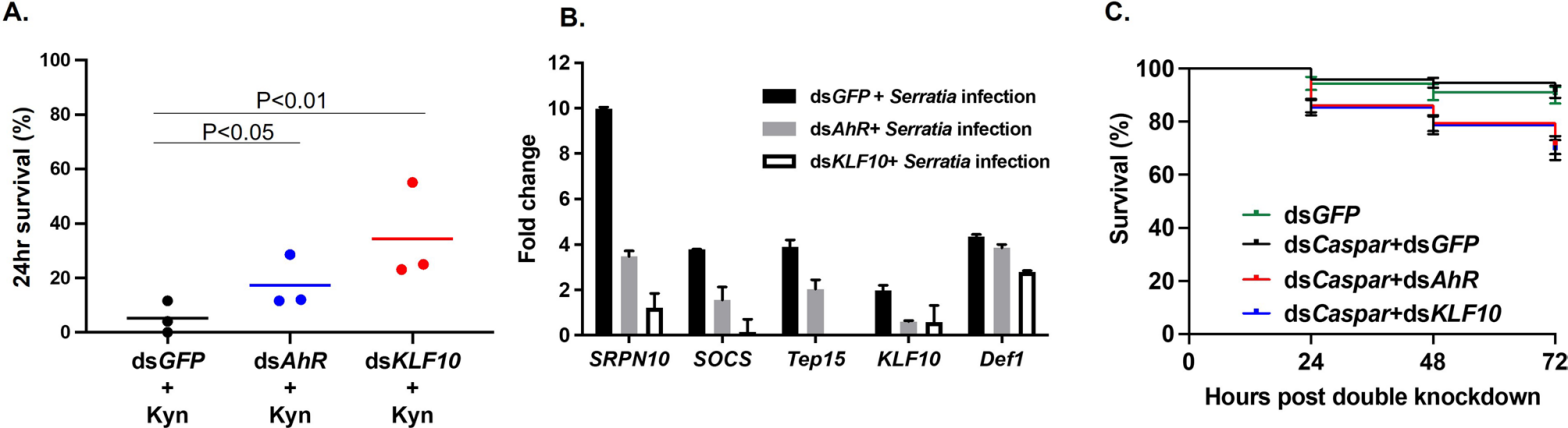
KLF10 is mediating immunomodulation downstream of AhR. (**A**) The Kyn-mediated immune suppression was reversed by dsAhR and dsKLF10, shown by the survival post bacterial challenge. (**B**) qPCR results of the genes in the dsAhR or dsKLF10 cohorts*.* (**C**) The effect of dsAhR and dsKLF10 on the survival of mosquitoes in which the IMD was overactivated by dsCaspar, no bacterial challenge was given. No significant difference was observed when dsCaspar + dsGFP was compared to dsGFP (Mantel–Cox test, P > 0.05). The survival was reduced in the co-silencing mosquitoes (Mantel–Cox test, P < 0.001). Each line represents pooled data from 5 cohorts with standard error bars.

Next, we reason that if the AhR activation mediated immune suppression requires KLF10, then the *KLF10* silencing would attenuate the immune suppression by AhR activation. Indeed, the reduced survival by Kyn feeding was reversed by dsKLF10 (P < 0.01, Fig. 6A). This result suggests that KLF10 acts downstream of AhR. The AhR–KLF10 axis regulates certain genes with potential immune regulatory functions. For example, the transcription of *SRPN10, TEP15, SOCS* was down-regulated by *AhR* and *KLF10* silencing in response to the bacterial challenge (Fig. 6B). These genes may have immune regulatory functions in the context (see “Discussion”).

### The AhR–KLF10 axis counteracts immune overactivation

From the observations above, we conclude that AhR and KLF10 constitute a signaling axis to modulate the immune response. This axis may help maintain immune homeostasis by counteracting adverse effects of immune overactivation. To test this hypothesis, we created an overactivated immune state by silencing *Caspar*, the negative regulator of IMD pathway^39^. *Caspar* was silenced by dsCaspar, and no bacterial challenge was given. The *Caspar* knockdown was confirmed by RT-PCR, and the transcript abundance of *Defensin* and *Cecropin* was elevated modestly as expected (Fig. S6), indicating the IMD pathway was activated. In this setting, the IMD pathway was active in a context without bacterial challenge. Presumably, this immune activation could come with a fitness cost. The fitness cost of the artificial IMD activation was measured by the post-treatment survival. Compared to the dsGFP cohort, survival of the dsCaspar cohort was not affected over a 72 h period (Mantel–Cox test, P > 0.05). However, when *AhR* or *KLF10* was co-silenced with *Caspar*, the survival was reduced (Mantel–Cox test, P < 0.001) (Fig. 6C). The results suggest that the presence of the AhR–KLF10 axis prevents the adverse effect of the overactivation of IMD without an insult.

## Discussion

AhR is conserved from invertebrate to vertebrate. During evolution, in addition to its ancestral role in the development of sensory structure and neural systems, AhR has diversified into a chemical sensor with binding affinity for a broad spectrum of intrinsic and/or extrinsic ligands derived from the environment or associated microbiota^40^. The ligand recognition transduces the chemical signals into responses in various contexts, such as xenobiotic detoxification and immune regulation in vertebrates. Tryptophan metabolites like kynurenine or bacteria-derived indoles produced by tryptophanase (tnaA) dependent tryptophan metabolism are known to be common AhR ligands^41^. In *C. elegans* and *D. melanogaster*, triggered by bacterial indoles, AhR directs a process to extend healthspan^12^. In this study, we demonstrate that mosquito AhR and KLF10 mediate a transcriptional axis that directs a transcriptional regulatory network in the immune context using a bacterial infection model. The immune outcome was measured by 24 hr survival upon a bacterial injection. AhR activation by agonists decreased the survival, while AhR inactivation by antagonists or the TDO inhibitor increased the survival (Fig. 1A). Corroborating the pharmacological evidence, silencing *AhR* increased survival as well (Fig. 1B). The AhR target genes in the context were screened by transcriptome interrogation. AhR manipulation did not affect transcription of the canonical antimicrobial effector genes, such as *Defensin* and *Cecropin*, suggesting that the AhR function is distinct from IMD and TOLL pathways. The infection inducible and AhR dependent genes include genes with possible immune-regulatory functions (Tables S3, S4). For example, *SRPN10*, *TEP15* and *SOCS* were AhR dependent in the context (Fig. 6B, Fig. S2). Serpins are protease inhibitors or molecular chaperones^42^. There are complex interactions between serine proteases and serpins in immune contexts^42,43^. SRPN10 gene is upregulated upon *P. berghei* infection, but its role in the immunity against *P.berghei* has not been elucidated^44,45^. It is reasonable to speculate that SRPN10 may regulatorily interact with target proteases to regulate immune responses. SOCS proteins are negative-feedback inhibitors of JAK-STAT pathway^46–48^. In *An. gambiae*, silencing of SOCS gene increases anti-*Plasmodium* immunity^48^. TEP family has 19 members in *An. gambiae*^43,49^, TEP1, TEP3, TEP4 have been characterized in different immune settings^50–52^. TEP15 has not been characterized at all. Based on the structure, TEP15 is annotated as the ortholog of CD109 in the α2-macroglobulins (A2M) subgroup in the TEP family (see Vectorbase). In mammals, CD109 plays a role as a negative regulator in TGF-β signaling pathway. These genes may have immune regulatory functions in the context.

This implies that AhR activation in the infection context may mediate the transcriptional regulations of a negative loop to maintain an immune balance. A variety of genes were affected by the AhR manipulations, including several transcription factors. We further characterized a zinc finger C2H2 type transcription factor, KLF10, which is the vertebrate ortholog of Krüppel like factor 10^53^. *Drosophila* and vertebrate KLF10 are involved in the TGF-β1 pathway^23,27^. TGF-β signaling plays a critical role in immune regulations in both invertebrates and vertebrates^54^. In *Drosophila*, upon injury and bacterial infections, two TGF-β signals in hemocytes are induced by respective cues, BMP (*dpp*) repressing antimicrobial peptide production, and Activin (*daw*) suppressing melanization^55^. Recently, AhR–TGF-β1/Smad signaling has been shown to mediate immune suppression in scurfy mice, a mouse autoimmune model^28^. In this study, we generated three lines of evidence to demonstrate that KLF10 is downstream of AhR to mediate immune regulation. First, *KLF10* silencing improved the survival (Fig. 2); second, the immune suppressive effect induced by the AhR activation via Kyn was reversed by dsAhR and dsKLF10, respectively (Fig. 6A); third, dsAhR and dsKLF10 resulted in similar transcriptional profiles, out of the 3285 dsAhR affected genes, 2245 (~ 68%) were altered by dsKLF10 as well (Fig. 5). The AhR–KLF10 axis mediates a systemic regulatory loop that sustains a homeostatic immune state. To corroborate this conclusion, we created an over-activation of IMD by depleting the negative regulator Caspar in naïve mosquitoes without a bacterial challenge. Sustaining IMD overactivation without an insult would be costly to fitness. As shown in Fig. 6C, the dsCaspar mediated IMD overaction without a challegne did not affect the survival when the AhR–KLF10 axis was present, however, the co-silencing of *Caspar* and *AhR* or *KLF10* significantly reduce the survival. Apparently, the AhR and KLF10 axis is required for the protection from the deleterious effect of IMD overactivation. Taken together, AhR and KLF10 mediate a transcriptional network to carry out immunoregulatory functions.

It is long known that vertebrate AhR mediates immunomodulation through its target genes^11^. However, many AhR target genes can also be regulated by other transcription factors, and it is challenging to characterize these genes in various immune contexts. In this study, KLF10 was identified as a major transcription factor downstream of AhR in response to a bacterial infection in mosquitoes. The transcriptome interrogation revealed a gene network that is controlled by the AhR–KLF10 axis. KLF10 connects energy metabolism and the circadian clock through the sugar sensing pathway in *Drosophila*^21,34,35^. This network appears to be active in the infection situation in mosquitoes, as the KLF10 target genes include the Mondo-Mlx dependent genes *pepck* as well as the circadian genes *tim, vri* and *cwo* (Fig. S5). Pepck converts oxaloacetate (OAA) to phosphoenolpyruvate (PEP), which is a rate-limiting step in gluconeogenesis, representing a critical link between biosynthesis and cataplerosis of the citric acid cycle^56,57^. Glycolysis and gluconeogenesis are delicately balanced as energy supply for fueling immune activities in neutrophils^58^. Evidence is mounting that metabolic homeostasis is critical for sustaining appropriate immune functions^59–61^, and KLF10 is a key player in metabolic regulation^62^. As the transcription factor downstream of AhR signaling, KLF10 is worth further investigations in mosquito immunometabolism. Interestingly, the axis restrains a large set of genes from responding to the challenge. When the axis was present, these genes did not transcriptionally respond to the challenge. However, when *AhR* and *KLF10* were silenced, their transcription went up or down. This indicates that the axis may control the transcriptional dynamics of these genes in the context. These genes may be transcriptionally chaotic without the axis. The annotations of these genes reveal diverse functions (Table S4), further investigation is warranted to decipher their roles in immune contexts.

In a recent study, it was shown that in *Aedes aegypti, An. stephensi and Culex quinquefasciatus,* spontaneous and induced mutations in the gene encoding *kynurenine hydroxylase* (KH) cause gut microbial dysbiosis and fitness abnormality^63^. In the kynurenine pathway of tryptophan degradation, KH catalyzes the hydroxylation of kynurenine to 3-hydroxyl-kynurenine. The mutation of *KH* leads to the accumulation of kynurenine and kynurenic acids. The heterozygous KH mutant *Aedes* mosquitoes experience a higher bacterial load in the sugar-fed or blood-fed guts. Similarly, the gut microbiota in the homozygous mutant *An. stephensi* increased ~ 800-fold after blood feeding^63^. In light of the current study, kynurenine is an endogenous AhR ligand, the elevated amount of kynurenine in the mutant mosquitoes may trigger and sustain an overactivation of AhR, which downregulates the immune surveillance capacity in the gut and results in the gut microbial expansion to a dysbiotic state. In addition, the persistent AhR activation in the mutant mosquitoes may create a transcriptional chaos. Uncontrolled expression of AhR target genes may contribute to gut dysbiosis and fitness deficiency in the mutant mosquitoes. These KH mutant mosquitoes can be used as valuable models to further study the function of AhR signaling in various mosquito traits.

In summary, we identified an AhR–KLF10 mediated transcriptional network that orchestrates immune modulations integrating various processes, including sugar sensing and central carbohydrate metabolism, pertinent to maintaining immune homeostasis. Further studies are required to identify AhR ligands that initiate the axis and elucidate the functional connection between energy metabolism and immune response in mosquitoes.

## Materials and methods

### Mosquitoes

*Anopheles gambiae* G3 strain was used for this study. The G3 mosquitoes were reared in an insectary with 28 °C and 80% humidity, a light/dark cycling of 14:10 h. Larvae were cultured in water pans with food (1:1 mix of the ground pellet of cat food and brewer’s yeast), and adults were maintained on 10% sucrose sugar meal, and fed on mice to acquire blood for egg production (The animal protocol was reviewed and approved by the NMSU IACUC).

### Phylogenetic inference using AhR protein sequences

The AhR protein sequences of representative organisms from invertebrates to mammals (Table S1) were used for inferring phylogenetic relationships. The sequences were aligned using the MUSCLE algorithm. A phylogenetic tree was made by the Neighbor-Joining method using Jones–Taylor–Thornton (JTT) model, with 500 bootstrap replications. Similar tree topology was generated by the Maximum Likelihood method using the JTT model with 500 bootstrap replications. Only the NJ tree is shown in Fig. S1. The domains were visualized in Simple Module Architecture Research Toll (SMART, http://smart.embl-heidelberg.de/), and the peptide sequences of domains bHLH, PAS A, and PAS B were extracted and protein sequence identity between these organisms were compared.

### Pharmacological manipulation of AhR activity in mosquitoes

AhR antagonists and agonists were used to manipulate AhR activity in mosquitoes. In each case, chemicals were fed to newly emerged adult mosquitoes, 50–60 females per cohort, in 10% sucrose solution for three days and then challenged with *Serratia fonticola* S1 (see below). Kynurenine (Kyn) is an endogenous ligand of AhR^14^. Kyn is generated during the oxidation of tryptophan, a reaction catalyzed by tryptophan-2,3-dioxygenase (TDO) in mosquitoes^15^. Kyn at 30 μM (Sigma-Aldrich, K8625) was used as an agonist to enhance the AhR activity. We used two AhR antagonists CH223191 at 90 μM (Sigma-Aldrich, C8124) and Stem Regenin (SR1) at 3 μM (Selleckchem, S2858). Additionally, we used 680C91 (20 μM) (Sigma-Aldrich, SML0287), a TDO inhibitor, to reduce endogenous Kyn production. The concentrations of these chemicals were chosen empirically based on the resulting phenotypes after administration.

### Bacterial infection

Bacterium *Serratia fonticola* S1 was isolated from a wild-caught specimen of *Aedes albopictus*, collected in Florida in July 2015. The bacteria were transformed with a plasmid expressing GFP as described previously^64^. The bacteria were grown overnight in Luria Bertani broth containing ampicillin (100 μg/ml) at 28 °C. The bacterial culture at OD_600nm_ = 1 was diluted 1000 times with sterile H_2_O, and approximately 100 nl of this bacterial solution yielded approximately 100 colony-forming units (CFU) per mosquito. As we have shown in a previous study^13^, the injection of this bacterial amount causes an acute bacterial infection, all mosquitoes die within three days post injections; thus, the 24 h-survival percent is the most informative data point to measure the infection outcome and the effects of treatments as well^13^. In this study, on day 3 after AhR activity was manipulated as described above, the mosquitoes were challenged with the bacteria, survival at 24 h post-infection was documented to examine the effects of AhR manipulation on the infection outcomes. The 24 h-survival percent between the treatment and respective control was tested using a Chi-square test. The data were generated from 5 experimental replicates except in Fig. 2D, where data were generated from 3 replicate experiments. For all injection experiments, post prior treatment, at least 30 mosquitoes survived to the point for bacterial challenge, which satisfies the statistical power.

### RT-PCR

Mosquito RNA was extracted from 15 females using Trizol reagent (Invitrogen, Cat# 15596026). Genomic DNA contamination was removed by DNaseI treatment using TURBO DNA-free Kit (Invitrogen, AM1906). The cDNA synthesis was carried out using NEB ProtoScript II Reverse Transcriptase (NEB, M0368S). The cDNA was used as a template for RT-PCR, to determine transcript abundance for various genes. The primers used are listed in Table S2. No reverse transcriptase (NRT) and no template control (NTC) served as negative controls.

### RNAi mediated gene knockdown in mosquitoes

RNAi-mediated *AhR*, *KLF10,* and *Caspar* knockdowns were performed. For dsRNA preparation, a target gene fragment was PCR amplified using gene-specific primers with the T7 promoter sequence at the 5’ end. The PCR products were used to synthesize dsRNA using a T7 RNA Polymerase Kit (Sigma-Aldrich RPOLT7-RO ROCHE). Generated dsRNAs were treated with TURBO DNA-free Kit (Invitrogen, AM1906) to remove DNA. The dsRNA of a GFP fragment was used as control dsRNA. Mosquitoes were injected with 0.5 μg/μl dsRNA to initiate RNAi. To co-silence *Caspar* along with either *AhR* or *KLF10*, respective dsRNA, each at 1.0 μg/μl, were mixed for injection. Newly emerged *An. gambiae* female mosquitoes, 60 females per cohort, were subjected to intrathoracic injections with ~ 100 nl of dsRNA. Treated mosquitoes were maintained on 10% sucrose solution for three days. For the bacterial challenge, cohorts of dsGFP control, dsAhR, and dsKLF10 were injected with *Serratisa* at day 4 post dsRNA treatment as described above. The gene knockdown efficacy was verified by RT-PCR with primers in Table S2.

### Transcriptome analysis

RNAseq was used to compare transcriptomes between samples with AhR manipulation. To examine the effect of pharmacological AhR inhibition, three cohorts of females were used. Mosquitoes were fed with AhR antagonist SR1 or vehicle control for three days, and then were challenged with *Serratia* infection, as described above. An injury control (in which mosquitoes were injected with sterile H_2_O) was included to control the effect of damage associated with the injection. To examine the effect of gene knockdown*,* three cohorts of newly emerged females were used; each was treated with dsAhR, dsKLF10, or dsGFP control, for 3 days, and then challenged with *Serratia* infection. Total RNA from 15 live mosquitoes at 24 h post-challenge was isolated using Trizol reagent, and then DNaseI treatment was followed to remove DNA contamination. Triplicate RNA samples were prepared for each treatment. The RNA samples were further processed at Genewiz, NJ, where the cDNA libraries were prepared and sequenced using Illumina Hiseq, 2 × 150 bp paired-end chemistry. At least 25 M clean reads were generated from each RNA sample, which provided a sequencing depth sufficient for transcriptome analysis. The reads were mapped against transcripts of *An. gambiae* (NCBI)*,* implemented by using Array Star v.12 (DNAstar). Read counts were normalized using the median of ratios method^65^ using DESeq2 software^66^. In determining normalized read counts, this method accounts for sequencing depth and RNA composition by calculating normalization factors for each sample in comparison to a pseudo-reference sample. After determining normalized read counts, an independent filter was utilized which removed transcripts with normalized counts less than 5. This resulted in a dataset of 10,714 transcripts. The clustering of all samples revealed that replicate 2 of dsAhR/*Serratia* infection was not consistent with the other two replicates, likely due to a quality issue associated with the replicate, therefore, this replicated was removed from the analysis. Differentially expressed genes were identified using a negative binomial generalized linear model (GLM) available through DESeq2^66^. Likelihood ratio tests were conducted to identify transcripts that exhibited differential expression between all groups. Pairwise differential expression comparisons were made and statistical significance was determined by computing q-values that preserve the False Discovery Rate (FDR)^65,67,68^. For example, concluding that a transcript was differentially expressed between two groups with a q-value of 0.05 would imply that there was a 5% chance (expected) that this conclusion was a false positive. To determine a lower-dimensional representation of the transcriptomic data, principal components analysis (PCA) was conducted using regularized log-transformed (rlog) data. PCA seeks to find a small set of “principal components” that capture a large proportion of the variance in the original data^69^. The rlog data was determined using DESeq2, while the “prcomp” function in R^70^ was utilized to determine the PCA. The proportion of the variance captured by each of the principal components was determined. Weighted gene co-expression network analysis (WGCNA)^71^ was conducted to identify modules (or sets) of transcripts that are co-expressed. This analysis was conducted as follows. First, the topological overlap between transcripts in a signed and soft-thresholded correlation network determined from the rlog data was determined using the R package WGCNA^72^. Further, transcript modules were determined utilizing hierarchical clustering with an “average” link function and utilizing the hybrid version of the “Dynamic Tree Cut” algorithm^73^. To validate expression patterns revealed by RNAseq, a selected set of genes was measured using quantitative RT- PCR. Primers were presented in Table S2. TPM (Transcripts Per kilobase Million) was used for the comparison of transcriptional abundance between different conditions^74^. The KEGG pathway analysis was implemented at Pathview Web, an online tool for pathway-based data integration and visualization^75^. The normalized read counts were used as input for Pathview analysis. The RNA-seq datasets are available at NCBI SRA under BioProject PRJNA691571.

## Supplementary Information

Supplementary Information.

Supplementary Table S1.

Supplementary Table S2.

Supplementary Table S3.

Supplementary Table S4.
