# Supplementary material for: Aryl hydrocarbon receptor and Krüppel like factor 10 mediate a transcriptional axis modulating immune homeostasis in mosquitoes
Source: Sci Rep. 2022 Apr 9;12:6005. doi: 10.1038/s41598-022-09817-2 (PMC8994780; doi:10.1038/s41598-022-09817-2)
Supplement: Supplementary file 1 — Supplementary Information. [file 41598_2022_9817_MOESM1_ESM.pdf]

Supplementary Figures and tables

Aryl hydrocarbon receptor and Krüppel like factor 10 mediate a transcriptional axis modulating immune homeostasis in mosquitoes

Aditi Kulkarni[1], Ashmita Pandey[1], Patrick Trainor[2], Samantha Carlisle[3], Wanqin Yu[1], Phanidhar Kukutla[1], Jiannong Xu[1*]

Jiannong Xu
Email: jxu@nmsu.edu

Figures S1 to S6

Original gel images of Fig.1B and Fig.2A insert

Table S1 to S2

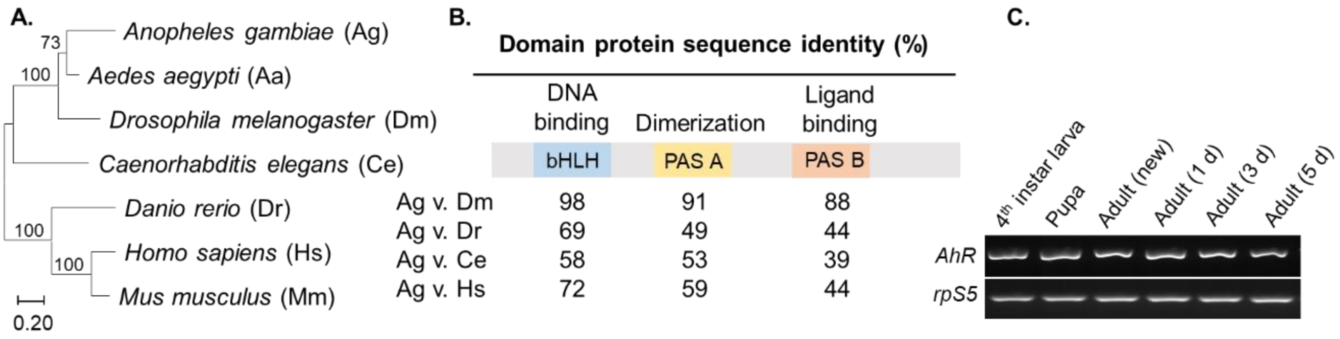

Fig. S1. **(A)** The AhR phylogenetic tree inferred from amino acid sequence comparison using Neighbor-Joining method. Bootstrap values were displayed on nodes. The scale bar indicates the genetic distance. **(B)** The level of conservation of domains bHLH, PAS A and PAS B, represented by the identity of the protein sequence. **(C)** *AhR* is constitutively expressed in larvae, pupae and adults (newly emerged, 1-, 3-, 5-day old), assayed by RT-PCR, *rpS5* was used as cDNA input control. The orinal gel image was attached below.

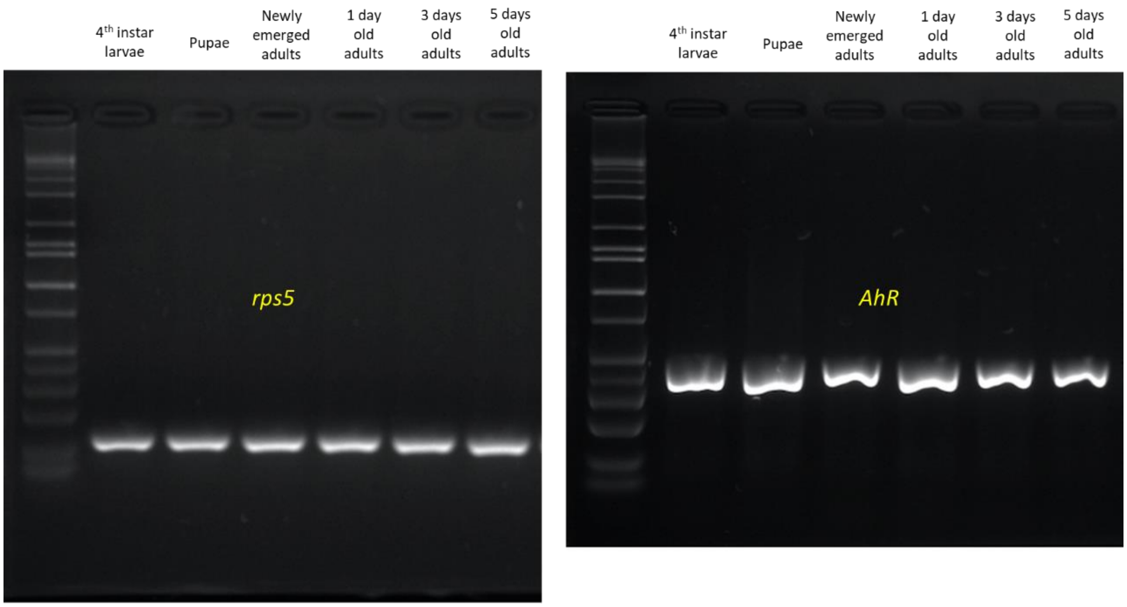

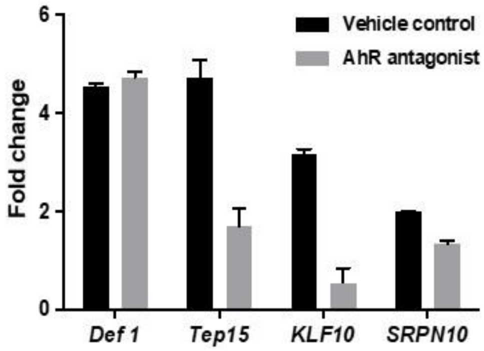

Fig. S2. Transcriptional validation of selected genes by qRT-PCR. Def1 represents an AhR independent immune gene, and *Tep15*, *KLF10* and *SRPN10* represent the AhR regulated genes. Fold change was calculated relative to injury control. Error bars represent SE.

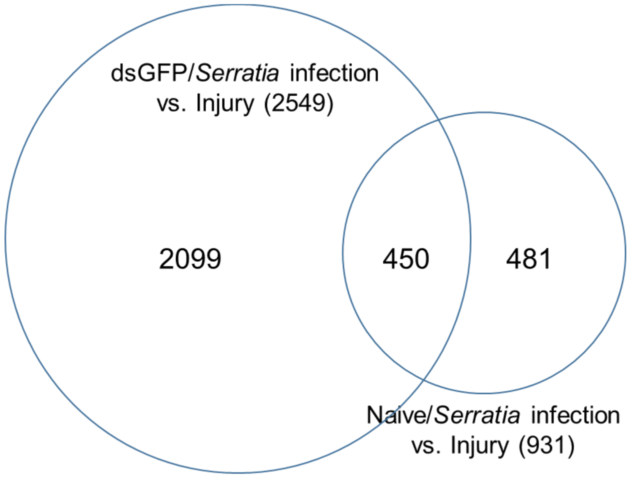

Fig. S3. Different responses to the *Serratia* challenge between the dsGFP and naïve cohorts. The dsGFP treatment altered 2.73-fold (2549/931) more genes than the naïve background, and only 450 genes were shared between.

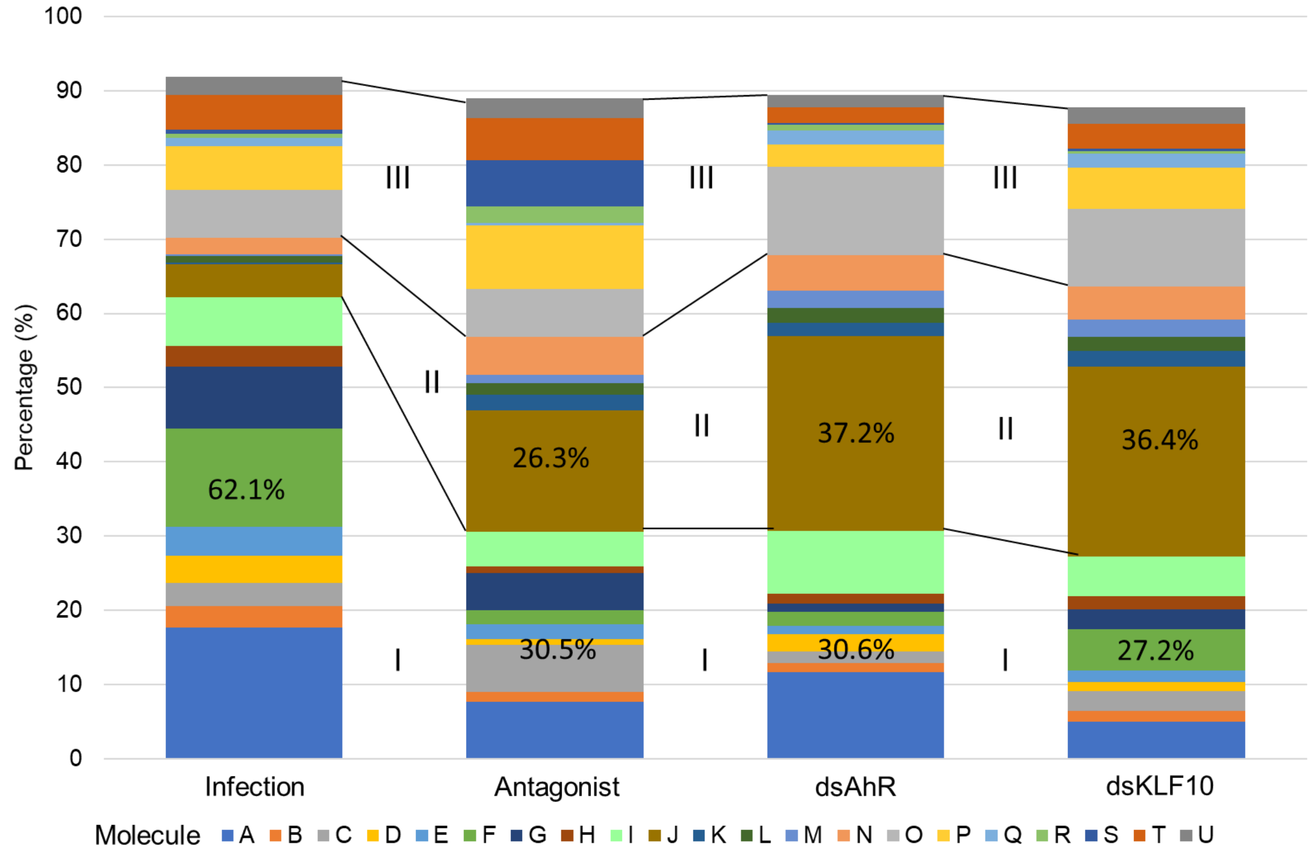

Fig. S4. Co-expression modules analysis. The co-expression pattern was similar between the AhR antagonist, dsAhR and dsKLF10 cohorts. The Category I modules contain more genes in the Infection cohorts than the AhR inactivated cohorts, while the Category II modules contain more genes in the AhR inactivated cohorts. The patterns represent the shared effects of AhR manipulation by both pharmacological and genetic approaches.

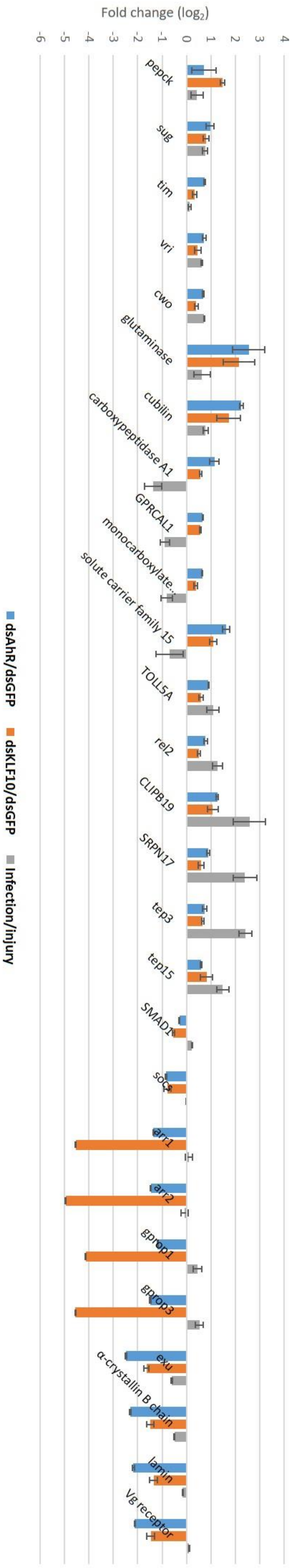

Fig. S5. The target genes of the AhR-KLF10 axis. The fold change ($\log_2$) was calculated based on the TPM values between the dsAhR or dsKLF10 and dsGFP cohorts upon the *Serratia* challenge. The fold change of infection/injury in the naïve background was presented as a reference. Bars represent standard error derived from three replicates.

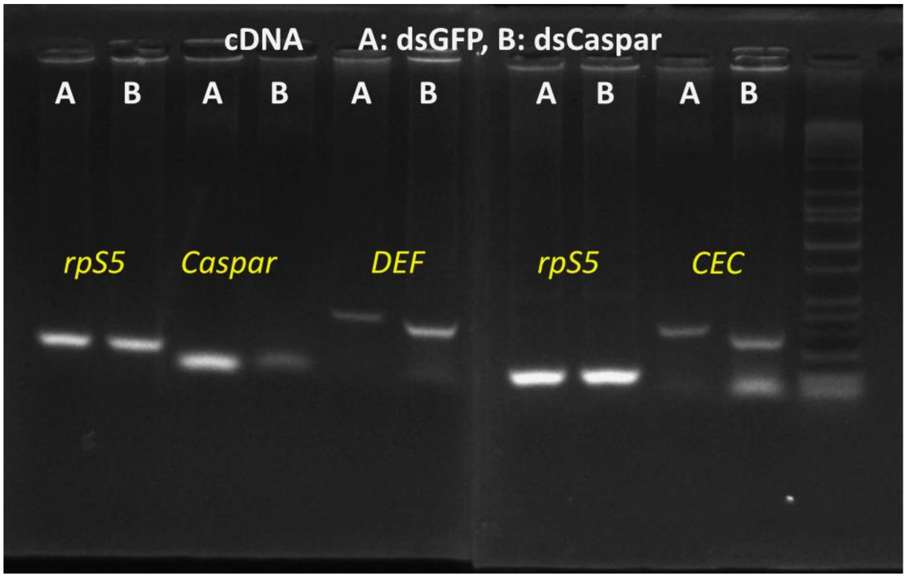

Fig. S6. Verification of dsCaspar knockdown by RT-PCR. Compared to the dsGFP cohort, dsCaspar resulted in the reduction of *Caspar* abundance, and *Def* and *Cec* both were upregulated as expected. The cDNA input was normalized by *rpS5*.

Original gel image of Fig. 1B

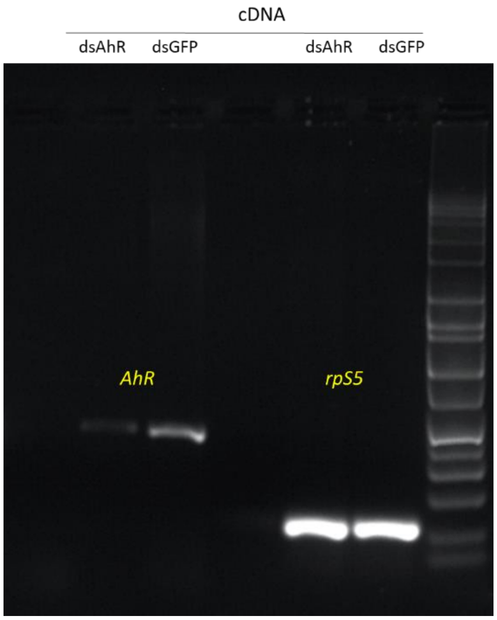

Orignial gel image of Fig. 2A insert

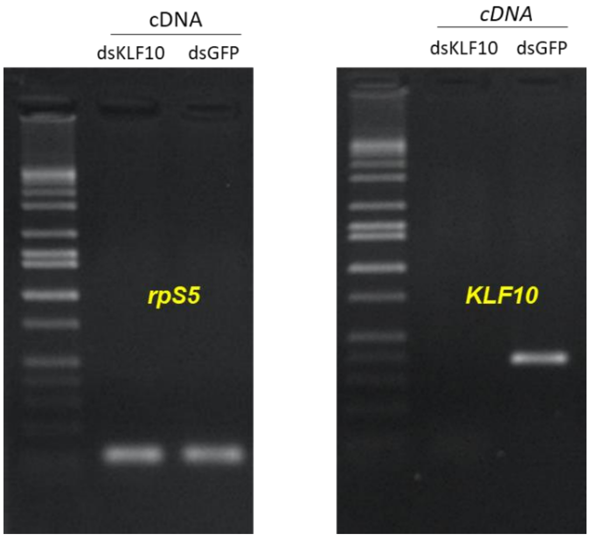

Table S1.

Table S1. AhR protein sequences used in phylogenetic analysis

Organism	GenBank accession
Aedes aegypti	EAT36061.1
Anopheles gambiae	XP_001238158.2
Drosophila melanogaster	NP_476748.1
Homo sapien	NP_001612.1
Mus musculus	NP_038492.1
Caenorhabditis elegans	NP_001021036.1
Danio rerio	NP_001019987.1

Table S2. Primers used in the study			
**Gene ID**	Gene name	**Forward**	**Reverse**
**AGAP005246**	SRPN10	GGGAGCGACATGACGATGAT	ACGGATGGTCTAGCCGTACT
**AGAP006473**	Caspar	CACCTGGAAGCATCGAACTGG	GTTGAGGTCGTTGGAGTACAGGC
**AGAP006473**	dsCaspar	TAATACGACTCACTATAGGGCCGCTTTTCTAAACGCTGTC	TAATACGACTCACTATAGGGAACCGTAATCCAAGGAGCA
**AGAP008329**	rps5	CCATGTCACGTCTCGTCACT	CGAAAACCATCCACACACAC
**AGAP008364**	TEP15	TCGAACGGTAAAGCTTCGCT	TCGAAGGAGCAGTCGAAACC
**AGAP009889**	KLF10	GCATCAACAAGGACGGTTCG	CTTGGGTATCACAGGAA
**AGAP009889**	dsKLF10	TAATACGACTCACTATAGTTCCTGTGATAGCGCCCAAG	TAATACGACTCACTATAGTTGTTGTGCCGTTTGACGTG
**AGAP010259**	AhR	AAACACAGCTGCGCGATTTT	CAGTACCGGGAAAGTGTGCT
**AGAP010259**	dsAhR	TAATACGACTCACTATAGGGAGGGGACAAAGAGCTACGG	TAATACGACTCACTATAGGGAAGCGGCCGGTGATTTTAC
**AGAP011294**	Def1	ATGCATTGTGCATTTGCCTA	AGCGGGACACAAAATTGAAC
**AGAP029624**	SOCS	GTTTTCCGTCTCCTTCCGCAAGTA	CTTCGGTAGCGTCAGCTCGTTGAT
	dsGFP	TAATACGACTCACTATAGAGTGAGCGCAACGCAATTA	TAATACGACTCACTATAGCAGCACGCGTCTTGTAGTTC

Table S3. Transcriptomic comparisons upon AhR and KLF10 manipulation followed by bacterial challenge. Fold change (log2), significance (q-values), expression modules, KEGG and Gene Ontology annotation are presented.

Table S4. Transcriptomic comparisons of the gene set (1617 genes) that are controled by both AhR and KLF10.
